# Liquid Contact-Selective Potentiometric Sensor Based on Imprinted Polymeric Beads Towards 17β-Estradiol Determination

**DOI:** 10.3390/polym12071506

**Published:** 2020-07-07

**Authors:** Ayman H. Kamel, Abd El-Galil E. Amr, Hoda R. Galal, Elsayed A. Elsayed, Ahmed I. Al-Sayady

**Affiliations:** 1 Department of Chemistry, Faculty of Science, Ain Shams University, 11566 Cairo, Egypt; ahkamel76@sci.asu.edu.eg; 2 Pharmaceutical Chemistry Department, Drug Exploration & Development Chair (DEDC), College of Pharmacy, King Saud University, Riyadh 11451, Saudi Arabia; 3 Applied Organic Chemistry Department, National Research Center, Dokki, 12622 Giza, Egypt; 4 Inorganic Chemistry Department, National Research Center, Dokki, 12622 Giza, Egypt; hrgalal@hotmail.com; 5 Bioproducts Research Chair, Zoology Department, Faculty of Science, King Saud University, Riyadh 11362, Saudi Arabia; eaelsayed@ksu.edu.sa (E.A.E.); 439105516@student.ksu.edu.sa (A.I.A.-S.); 6 Chemistry of Natural and Microbial Products Department, National Research Centre, Dokki, 12622 Cairo, Egypt

**Keywords:** potentiometry, man-tailored mimics, MIPs, 17β-estradiol, biological fluids

## Abstract

Novel potentiometric devices “ion-selective electrodes (ISEs)” were designed and characterized for the detection of 17β-estradiol (EST) hormone. The selective membranes were based on the use of man-tailored biomimics (i.e., molecularly imprinted polymers (MIPs)) as recognition ionophores. The synthesized MIPs include a functional monomer (methacrylic acid (MAA)) and a cross-linker (ethylene glycol dimethacrylic acid (EGDMA)) in their preparation. Changes in the membrane potential induced by the dissociated 17β-estradiol were investigated in 50 mM CO_3_^2−^/HCO_3_^−^ buffer solution at pH 10.5. The ion-selective electrodes (ISEs) exhibited fast response and good sensitivity towards 17β-estradiol with a limit of detection 1.5 µM over a linear range starts from 2.5 µM with an anionic response of 61.2 ± 1.2 mV/decade. The selectivity pattern of the proposed ISEs was also evaluated and revealed an enhanced selectivity towards EST over several phenolic compounds. Advantages revealed by the presented sensor (i.e., wide range of assay, enhanced accuracy and precision, low limit of detection, good selectivity, long-term potential stability, rapid response and long life-span and absence of any sample pretreatment steps) suggest its use in routine quality control/quality assurance tests. They were successfully applied to estradiol determination in biological fluids and in different pharmaceutical preparations collected from the local market.

## 1. Introduction

17β-Estradiol is a natural estrogen belonging to the natural steroidal hormones. This class of hormones is essential in the reproductive processes of females and decisively affecting mammal fertilization. In addition, it controls numerous physiological actions, especially in women. Some of these are body growth, menstruation, minerals, carbohydrates, protein, and fat metabolism. It also has an important role in males including in bone and sperm formation [1,2,3]. Low concentrations of estradiol in the human body can lead to developmental abnormalities and damage to the male reproductive system [4]. 17β-Estradiol poses a real risk to children in early puberty and also increases the risk of breast and ovarian cancer in women [5]. Some other diseases are related to this hormone, such as bladder cancer [6] and Alzheimer’s disease [7,8]. Mammals secrete this hormone and its derivatives daily through urine, which reach the wastewater in addition to remnants from the pharmaceutical industry. The presence of this hormone in the environment causes severe complications and shows high toxicity even in small amounts. It also interferes with the reproduction and development of fauna. Estradiol stimulates hormone production in mammals and changes its natural concentration in the bloodstream in addition to affecting metabolic processes [9]. Moreover, there have been reports that birds feeding on fish containing this hormone are vulnerable to their own immune system [10]. Accordingly, finding a reliable, sensitive, selective, and fast evaluation method for estradiol assessment in living organisms, food, or the environment is of the utmost importance.

There are a number of highly sensitive methods for detecting estradiol in the literature, such as high performance liquid chromatography (HPLC) [11,12], gas chromatography/mass spectrometry (GC–MS) [13,14], liquid chromatography/mass spectrometry (LC–MS) [15,16], immunoassay [17,18] and electrochemical methods [19,20,21,22,23]. However, the abovementioned methods have several drawbacks, such as the long sample preparation time, the high cost of the reagents used, and the requirement for well-trained personnel. The use of potentiometric sensors in the analysis enables them to get rid of these defects. These types of electrochemical sensors deserve particular attention because of their simplicity and their enhanced sensitivity of measurements [24,25,26,27,28,29].

Molecularly imprinted polymers (MIPs) have high affinity toward the target analyte. This high affinity is due to the presence of the pre-defined specific recognition cavities present in the skeleton of the MIPs. These biomimics have high stability towards pH changes, organic solvents and temperature. These advantages provide great flexibility in developing chemical and biological analysis methods [24,25,26,30,31]. Ion-selective electrodes (ISEs) based on the man-tailored imprinted polymers, MIPs, now demonstrate great attention for changing the way of using non-available ionophores [32,33,34,35,36]. Additionally, the membrane potential developed in ISEs does not require the template to be extracted from the skeleton of the synthesized MIP. There are also no size restrictions on the mold compound because the species should not diffuse across the membrane. Different MIPs based on MAA and EGDMA were prepared and reported for selective recognition of 17β-estradiol [37,38,39]. 

In this work, we report for the first time cost-effective, reliable and robust potentiometric ISEs for 17β-estradiol. Man-tailored biomimics for EST based on template imprinted polymers were synthesized using thermal precipitation polymerization and methacrylic acid (MAA) as an appropriate monomer. The proposed ISEs revealed a high sensitivity and selectivity for potentiometric monitoring of 17β-estradiol. The sensors were successfully applied for 17β-estradiol determination in urine and pharmaceutical formulations collected from the local market.

## 2. Experimental

### 2.1. Reagents and Apparatus

All aqueous solutions used in this work were prepared using de-ionized water (conductivity < 0.1 µS cm^−1^, Millipore Milli-Q Direct-0.3 purification system). Poly(vinyl chloride) (PVC), 2-nitrophenyloctyl ether (*o*-NPOE), dioctylphthalate (DOP) and dibutylsebacate (DBS) were obtained from Fluka AG (Buchs, Switzerland). 17β-Estradiol and tetradodecylammonium tetrakis (4-chlorophenyl) borate (ETH500) were purchased from Sigma Chemicals Co. (St. Louis, MO, USA). Na_2_CO_3_, NaHCO_3_, NaOH and NaCl were obtained from Acros.

A 50 mm HCO_3_^−^/CO_3_^2−^ buffer solution of pH 10.5 was used for all measurements to make sure that 17β-estradiol is in its ionized form. For the potentiometric selectivity study, a 1.0 × 10^−2^ M solution for each interfering ion was also prepared using 50 mM HCO_3_^−^/CO_3_^2−^ buffer solution, pH 10.5.

### 2.2. Man-Tailored Biomimics Synthesis

Man-tailored biomimics or MIPs were prepared using the thermal precipitation polymerization method. In brief, 3.0 mmol of cross-linked MAA monomer with 3.0 mmol of EGDMA was mixed with 1.0 mmol of EST as a template. The mixture was mixed together and then dissolved in 15 mL acetonitrile. BPO (80 mg) was added to the reaction mixture as an initiator. The cocktail was added in a 25 mL sealed tube. Nitrogen gas stream was diffused into the cocktail solution for 15 min for complete removal of dissolved oxygen. The tube was inserted in a paraffin wax at 70 °C for 20 h for complete polymerization. Non-imprinted polymer (NIP) particles were prepared by the same process but without using template molecule. The resulting powders were washed after drying with absolute ethanol several times in a Soxhlet extractor for 48 h. MIPs and NIPs were left to fully dry at room temperature before use.

### 2.3. Sensor Design and Potential Measurements

The membrane-based sensor was prepared by dissolving 64 mg PVC, 126 mg of the chosen plasticizer, 12 mg of MIP or NIP particles, and 1.4 mg ETH 500 in 3 mL THF. The cocktail was inserted into a glass cup (30 mm i.d.) and left overnight until complete evaporation of THF. A disk of about 6 mm in diameter was then cut and glued by THF to a piece of Tygon tube (5 mm in inner diameter, 9 mm in outer diameter and 2 cm in length). The Tygon tube was attached to a plastic electrode body, and a 1:1 mixture of 10^−2^ M NaCl solution and 1 mM 17β-estradiol solution (buffered with 50 mM HCO_3_^−^/CO_3_^2−^ buffer solution, pH 10.5) was used as internal filling solution. An internal reference electrode made from Ag/AgCl was inserted in the filling solution for electrical connection. The potential response versus an external Ag/AgCl double junction reference electrode was recorded.

The prepared ISEs were inserted for 6 h in 1 mM of EST solution (pH 10.5) for conditioning. Test solution was kept at pH 10.5 using 50 mM HCO_3_^−^/CO_3_^2−^ buffer solution. The potential of the solutions was recorded over different concentration range of EST solution to construct the calibration plot. 

### 2.4. Estradiol Assessment

The applicability of the proposed ISEs was tested in different urine samples. A 10 mL aliquot of different urine samples was transferred to a 100 mL measuring flask and then diluted to the mark using 50 mM HCO_3_^−^/CO_3_^2−^ buffer solution, pH 10.5. A 1.0 mL aliquot of EST solution to cover the range from 2 to 10 µM was transferred to a 20 mL beaker containing 9 mL of 50 mM HCO_3_^−^/CO_3_^2−^ buffer solution, pH 10.5. The sensor was immersed in conjunction with the reference electrode in the test solution. Possible readings were recorded after the equilibrium response was reached and compared with the calibration graph. 

Estradiol was also analyzed using the proposed ISEs in different commercially available drugs: Estraderm (50 mg/tablet, Novaris pharmaceuticals, Cairo, Egypt) and Oestrogel (0.06 % *w/w*, Gel, Besins). To examine estradiol in tablet formulations, 3 tablets were ground in an agate mortar. A specified amount of 3 finely mixed powder disks, equivalent to one tablet, was transferred to a 100 mL volumetric flask and dissolved in 20 mL aqueous NaOH solution (0.1 M), sonicated for 45 min. The solution is then adjusted to pH 10.5 with 50 mM HCO_3_^−^/CO_3_^2−^ buffer solution and then supplemented to the mark. Possible measurements of these solutions were carried out and the potential readings were recorded and compared to the constructed calibration plot. The preparation of the gel sample was followed by weighting the appropriate amount of the drug as well as dissolving it in a solution of 20 mL aqueous NaOH solution (0.1 M).

## 3. Results and Discussions

**Figure 1:**
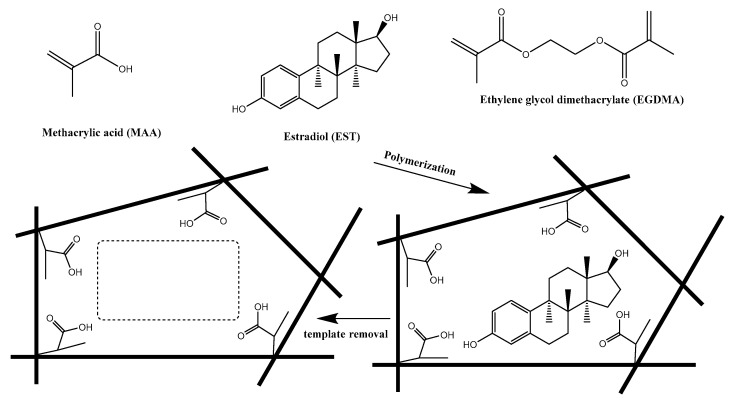
Protocol of synthesis of molecularly imprinted polymers (MIPs) and its recognition towards 17β-estradiol.

Herein, we presented, for the first time, a simple and sensitive analytical system based on MIPs for the assessment of 17β-estradiol (EST). For this purpose, we introduced an electrochemical sensor utilizing the potentiometric transduction of bound EST to the MIPs by electrochemical reaction. A schematic illustration for the molecular imprinting process is shown in Figure 1.

### 3.1. SEM Analysis of Biomimic Particles

**Figure 2:**
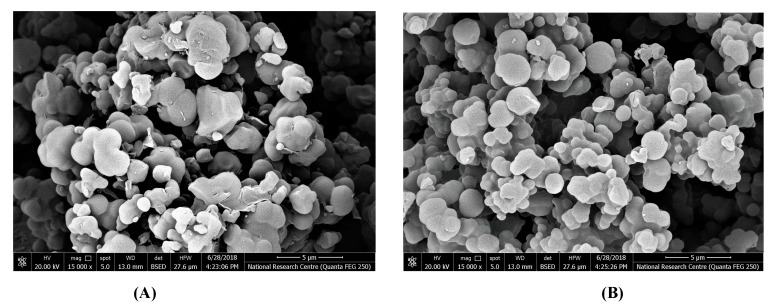
Scanning electron microscope (SEM) images of (**A**) non-imprinted polymer (NIP) beads and (**B**) washed MIP beads.

The morphological forms of both MIP and NIP surfaces were examined using an electronic scanning microscope (SEM) (Figure 2). Figure 2A presents a medium uniformity with a spherical shape for the NIP particles with an average diameter of about 1.8 µm. For the MIP particles, the surface morphology presented in Figure 2B showed irregular beads with a mean diameter of 0.7 µm. The different surface morphologies between MIP and NIP particles confirm the tracing of the printing process that ensures the MIP efficiency as a suitable ionophore for the recognition of estradiol in the presented sensors.

### 3.2. Potentiometric Detection of EST

**Figure 3:**
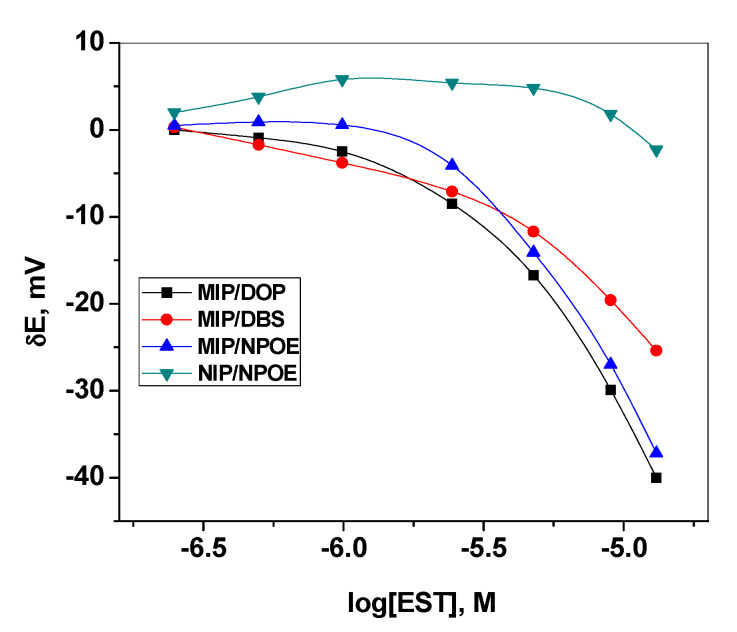
Effect of plasticizer type on the membrane response.

**Figure 4:**
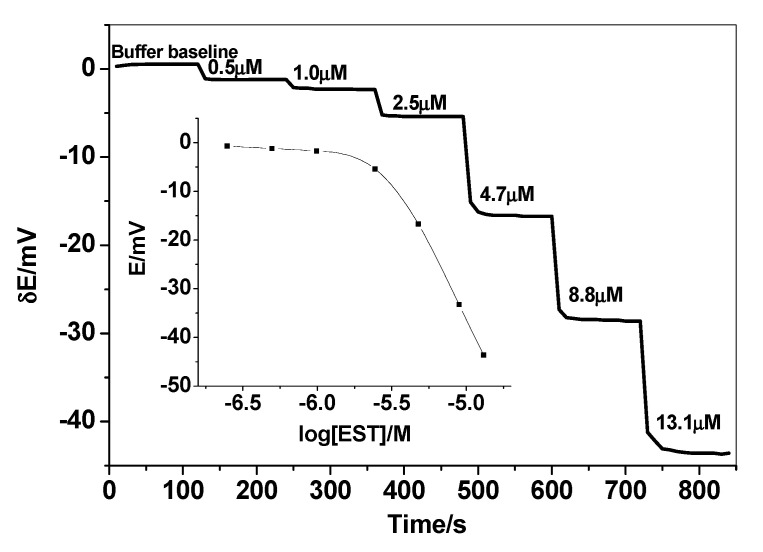
The dynamic potentiometric responses of 17β-estradiol (EST)-based sensor in 50 mM HCO_3_^−^/CO_3_^2−^ buffer solution, pH 10.5. The inset shows the measuring calibration plot for EST.

**Figure 5:**
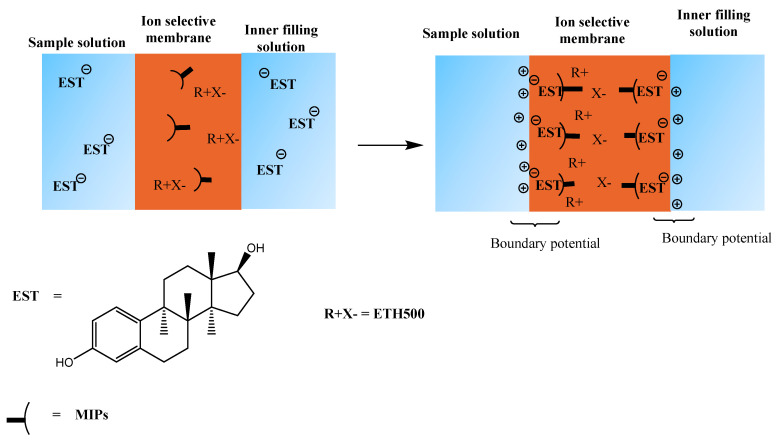
Response mechanism of the proposed 17β-estradiol sensor.

A novel PVC membrane sensor based on a newly synthesized MIP particles as a sensory recognition material, dibutylsebacate (DBS), dioctylphthalate (DOP) or *o*-nitrophenyloctyl ether (*o*-NPOE) as a solvent mediator, and PVC as polymeric matrix was prepared and tested as an ion sensor for detecting 17β-estradiol. The polarity of the membrane solvent not only affects the dissolution of the ionophore in the membrane but also can affect the movement of the ion in the membrane phase. Hence, membrane optimization should be considered in this study. Different plasticizers with different polarities were investigated and their influences on the potential response of the sensing membrane were recorded. As shown in Table 1, a membrane incorporating *o*-NPOE plasticizer (high dielectric constant, ε = 24) showed the best characteristics. The sensors display a linear response starts from 2.5 µM with an anionic response with a slope of 61.2 ± 1.2 mV/decade and a detection limit of 1.5 µM (3σ). As shown in Figure 3, better response behavior and better sensitivity was obtained with the polar plasticizer *o*-NPOE. This can be explained on the basis that EST prefers the high polar solvent to be distributed into the sensing membrane. The potential response of the proposed sensor towards EST is shown in Figure 4. The potential difference between baseline potential and those measured at a specific time (i.e., 120 s) was used after the addition of the EST for quantitative analysis. The presented sensor revealed fast response and stable potential. As a control, sensors based on NIP beads were also tested. These sensors possessed a linear range starts from 10 µM with a slope of −15.6 ± 1.5 mV/decade (R^2^ = 0.991) and a detection limit of 8.5 μg/mL. The sensing mechanism of 17β-estradiol using MAA- and EGDMA-based MIPs is illustrated in Figure 5.

### 3.3. Sensor Selectivity

**Figure 6:**
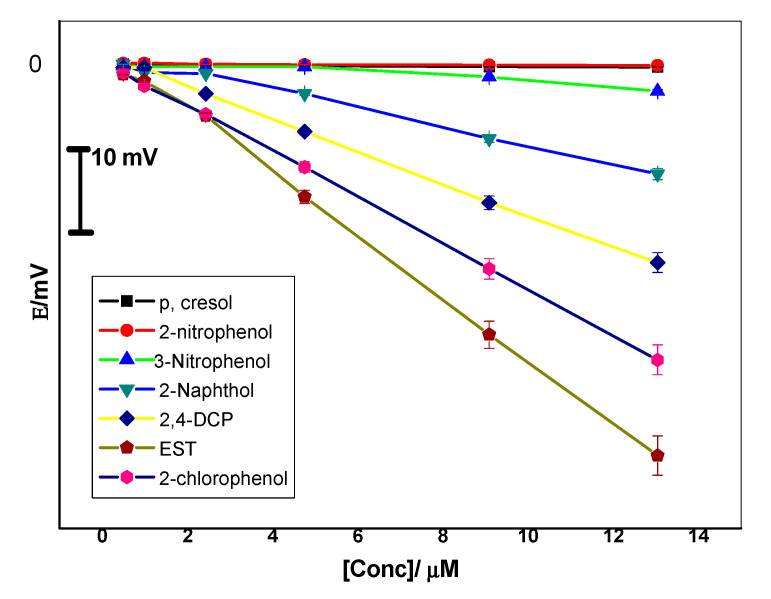
Potentiometric selectivity of MIP membrane-based sensors towards 17β-estradiol (EST).

The selectivity coefficient values of the proposed sensors were evaluated using the so-called “modified separate solution method (MSSM)” [40]. The potential responses towards EST were recorded as shown in Figure 6. The pKa values for phenol derivatives used in selectivity measurements lie in the range 7.8–10.5. Hence, pH 10.5 is the selected value to ensure the presence of the ionized form of these compounds. Experiments have shown that the selectivity arrangement of the MIP-based sensor is EST > 2-chlorophenol > 2,4-dichlorophenol > 2-naphthol > 3-nitrophenol > 2-nitrophenol > p-cresol. The selectivity order of these neutral phenols reflects their acidity and lipophilicity [41]. As the acidity and lipophilicity increases, the anionic response increases. Partition coefficients and acid dissociation constants of EST, 2-chlorophenol, 2,4-dichlorophenol, 2-naphthol, 3-nitrophenol, 2-nitrophenol and p-cresol are and 4.01, 2.15, 3.06, 2.7, 2.0, 1.79 and 1.94, and 10.07, 8.52, 7.89, 9.5, 8.3, 7.23, and 10.3, respectively [41].

### 3.4. Analytical Applications

The applicability of the proposed ISEs for EST determination was checked in urine samples.

However, sample dilution can be used to avoid the effect of the matrix. Adsorption of urine protein onto the PVC membrane in the sensors leads to poor reproducibility. Determination of EST was performed in biological fluids using the proposed estradiol probes. The potential responses of urine samples in 30mM PBS buffer of pH 7.0 and containing fixed concentration of estradiol were measured directly. The results showed an average mean recovery of 94.0–101.2% (Table 2) indicating minimal interference effect due to the matrix. Estradiol was determined using the standard addition method in two commercially available medicines collected from the domestic market. The results obtained with measured recovery for each drug are presented in Table 3. A measured recovery between 90 and 102.4% indicates that the proposed method for determining estradiol using the displayed electrode is appropriate for pharmaceutical analysis. From the obtained results by the proposed potentiometric method, it was compared to those obtained by the HPLC method [42]. The results of the *t*-Student and *F*-test confirmed that there were no statistically significant differences between the results of the two methods and revealed the successful application of the proposed ISE as a new analytical method for determining EST.

## 4. Conclusions

A reliable, robust, and cost-effective potentiometric sensor based on man-tailored mimics for the potentiometric transduction of estradiol has been presented. The MIP particles are dispersed into a plasticized PVC membrane. The ISEs displayed extended linear response range starts from 2.5 µM, low detection limit 1.5 µM and fast response time (<10 s). The presented electrodes revealed good advantages over many of those previously described in terms of durability, ease of manufacture, potential stability, selectivity, and accuracy. The proposed liquid contact estradiol-sensor was successfully used for trace determination of 17β-estradiol in different pharmaceutical formulations and urine samples. No sample pretreatment is required for estradiol analysis using these proposed ISEs.

## Figures and Tables

**Table 1:** Performance characteristics of 17β-estradiol PVC membrane sensor in 50 mM HCO_3_^−^/CO_3_^2−^ (pH 10.5).

Parameter	MIP/o-NPOE	MIP/DOP	MIP/DBS
**Lower limit of linear range, µM**	2.5	4.8	6.2
**Slope, mV/log [EST]**	−61.2 ± 1.2	−52.6 ± 2.2	−31.6 ± 1.8
**Low detection limit, µM**	1.5	3.2	4.3
**Correlation coefficient, r^2^**	0.9995	0.991	0.993
**Response time, s**	<10	<10	<10
**Accuracy, %**	99.3	98.7	97.3
**Trueness, %**	99.5	99.1	97.2
**Bias, %**	0.4	0.6	1.3
**Within-day repeatability, CV_w_ %**	1.1	0.9	1.4
**Between-days variation, CV_b_ %**	1.2	0.7	1.2

**Table 2:** Determination of EST in spiked urine samples using *o*-NPOE plasticized membrane sensors.

Sample	EST Added, µM		
	Added	Found *	Recovery, %
**1**	2.5	2.4 ± 0.3	96
**2**	5	4.7 ± 0.5	94
**3**	8	8.1 ± 0.8	101.2

* Average of 5 measurements.

**Table 3:** EST determination in pharmaceutical preparations using EST membrane sensor.

Pharmaceutical Product and Source	Nominal Content is Taken	Found				*t*-Student Test	*F*-Test
		Proposed Method	Mean ^a^ (%) ± SD	Reference Method [42]	Mean ^a^ (%) ± SD		
**Estraderm tablets, (Novartis, Egypt)**	50 mg/tablet	51.2	102.4 ± 0.7	50.2	100.4 ± 1.1	1.6	4.9
**Oestrogel gel (Besins)**	0.06 % *w*/*w*	0.054	90.0 ± 1.3	0.059	98.3 ± 0.9	1.5	5.2

^a^ Mean of three replicate measurements ± standard deviation (SD). *t*-Student and *F*-test at 95% confidence level values are 4.30 and 19.00, respectively.
